# Gene expression profiling identifies distinct molecular signatures in thrombotic and obstetric antiphospholipid syndrome

**DOI:** 10.1016/j.jaut.2018.07.002

**Published:** 2018-09

**Authors:** Vera M. Ripoll, Francesca Pregnolato, Simona Mazza, Caterina Bodio, Claudia Grossi, Thomas McDonnell, Charis Pericleous, Pier Luigi Meroni, David A. Isenberg, Anisur Rahman, Ian P. Giles

**Affiliations:** a Centre for Rheumatology Research, Division of Medicine, University College London, Department of Medicine, Rayne Institute, 5 University Street, London, UK, WC1E 6JF, UK; b Immunology Research Laboratory, IRCCS Istituto Auxologico Italiano, Via Zucchi, 18, 20095 Cusano milanino MI, Italy

**Keywords:** Gene expression profiling, Monocytes, APS, Vascular thrombosis, Pregnancy morbidity

## Abstract

Antiphospholipid antibodies (aPL) cause vascular thrombosis (VT) and/or pregnancy morbidity (PM). Differential mechanisms however, underlying the pathogenesis of these different manifestations of antiphospholipid syndrome (APS) are not fully understood. Therefore, we compared the effects of aPL from patients with thrombotic or obstetric APS on monocytes to identify different molecular pathways involved in the pathogenesis of APS subtypes. VT or PM IgG induced similar numbers of differentially expressed (DE) genes in monocytes. However, gene ontology (GO) analysis of DE genes revealed disease-specific genome signatures. Compared to PM, VT-IgG showed specific up regulation of genes associated with cell response to stress, regulation of MAPK signalling pathway and cell communication. In contrast, PM-IgG regulated genes involved in cell adhesion, extracellular matrix and embryonic and skeletal development. A novel gene expression analysis based on differential variability (DV) was also applied. This analysis identified similar GO categories compared to DE analysis but also uncovered novel pathways modulated solely by PM or VT-IgG. Gene expression analysis distinguished a differential effect of VT or PM-IgG upon monocytes supporting the hypothesis that they trigger distinctive physiological mechanisms. This finding contributes to our understanding of the pathology of APS and may lead to the development of different targeted therapies for VT or PM APS.

## Highlights

• Gene expression analysis distinguished a differential effect of VT or PM IgG upon monocytes.
• VT and PM trigger the expression of distinctive molecular pathways with functional relevance to these APS subtypes.
• These pivotal findings could enable the design of therapies targeted to the pathogenesis of different APS subtypes.

## 1 Introduction

The antiphospholipid syndrome (APS) is an autoimmune disease in which antiphospholipid antibodies (aPL) cause vascular thrombosis and/or pregnancy morbidity. Current APS classification criteria identify the presence of aPL using: anti-cardiolipin antibodies (aCL); anti-beta 2-glycoprotein I antibodies (aβ2GPI); and/or Lupus anticoagulant (LA) assays. The classification criteria for APS identify two major types of clinical events: thrombosis and pregnancy morbidity. Thus, there are three groups of patients with APS – those with a history of vascular thrombosis but not pregnancy morbidity (VT+/PM-), those with pregnancy morbidity but no vascular thrombosis (VT-/PM+) and those who have suffered both types of clinical events (VT+/PM+). Long-term follow-up studies over many years showed that most VT+/PM- patients never develop PM and most VT-/PM+ patients never develop VT [1,2] suggesting that, in these patients, the aPL that cause VT and those that cause PM may bind different antigens and cellular receptors to have diverse effects on target cells.

Many studies have aimed to determine whether specific aPL are associated with thrombotic or obstetric manifestations. One particular study reported LA as the primary predictor of adverse pregnancy outcome [3]. However, no one aPL test has emerged clearly as the leading marker of vascular thrombosis or pregnancy morbidity in APS.

A few studies found differences in the effects of aPL from patients with and without thrombosis upon various signalling pathways in target cells [[4], [5], [6], [7]]. Proteomic analyses of monocytes isolated from patients with APS [8] and healthy monocytes treated with APS-IgG [9] have shown that the monocyte proteome is differentially regulated by IgG from obstetric compared to thrombotic APS.

A limited number of microarray studies in APS have reported differences in gene expression in peripheral blood mononuclear cells (PBMCs) from patients with APS compared to healthy controls (HC) [10]; endothelial cells treated with aPL [11] and monocytes isolated from patients with APS, HC and/or SLE [12]. These studies however, did not examine for differences in gene expression between APS sub-types.

In the present study, we carried out microarray analysis comparing patterns of mRNA expression in monocytes from a healthy volunteer exposed to VT+/PM-, VT-/PM+ or HC-IgG. We specifically sought to compare the effects of aPL from patients with different manifestations of APS on monocytes.

Our microarray approach involved the traditional methods of analysis including the identification of genes with significant changes in mean expression level, also known as differentially expressed genes (DE). DE genes were defined as transcripts showing an unusually high or low expression level under a particular treatment compared with transcript expression levels of other genes under the same treatment. The DE genes of particular interest in this study are those which differ between the groups i.e. they are differentially regulated in monocytes treated with either VT+/PM- IgG or VT-/PM+ IgG but not both.

We also employed a novel form of analysis that looks at differential variability (DV) of genes. This analysis is not concerned with the absolute level of expression of particular genes, but the degree to which that level varies amongst cells exposed to different IgG samples from patients of the same phenotype. In the first description of the DV method, Ho et al. [13] argued that DV analysis was biologically relevant and valuable because it gives insight into cellular regulation. DV analysis typically identifies a smaller number of genes than DE analysis and may identify different genes than DE, so that the two types of analysis can be used in parallel as we have done in this paper. We have used both DE and DV analysis of gene expression data obtained by microarray to identify molecular signals and functional pathways that differ between thrombotic and obstetric APS.

## 2 Methods

### 2.1 Patients

Serum samples (n = 27) were obtained with informed consent and appropriate local ethical approval. Of 18 patients fulfilling APS classification criteria [14], 3 also had SLE, fulfilling the classification criteria [15] and 15 had primary (P)APS. Healthy individuals (n = 9) were aPL and APS negative.

### 2.2 Immunological characterisation and purification of IgG

IgG was protein G purified, passed through Endotoxin removal columns (Thermo Scientific) and confirmed to be < 0.06 endotoxin units/ml by EndoLISA^®^ (Hyglos). Concentration was determined by spectrophotometry. IgG aCL and anti-β2GPI titres were determined as previously [16]. Serum LA was measured by dilute Russell's viper venom time.

### 2.3 Isolation and culture of human monocytes

In order to reduce sample variability, peripheral venous blood samples from a single healthy donor were used to isolate mononuclear blood cells using SepMate tubes (StemCell Technology) and Ficoll-Paque Plus (GE Healthcare). Monocytes were purified using the immunomagnetic Easysep human CD14 + ve selection protocol (StemCell Technology). Monocytes were cultured at 37 °C and 5% CO_2_ in RPMI 1640 supplemented with 10% heat-inactivated FBS, 20 U/mL penicillin, 20 μg/mL streptomycin and 2 mM l-glutamine.

### 2.4 In vitro exposure of monocytes to IgG

For microarray hybridisation, 1 × 10^6^ monocytes were treated with 200 μg/ml of individual IgG from 8 VT+/PM−, 6 VT−/PM+ or 8 HC for six hours. For target validation, 2.5 × 10^5^ monocytes were treated with 200 μg/ml of individual IgG from 9 VT+/PM−, 9 VT−/PM+ or 9 HC for six hours. All IgG samples from APS displayed a higher aCL (>40 GPLU) and anti-β2GPI (>10 GBU) binding compared to healthy controls that were negative in these assays.

### 2.5 RNA extraction, labelling and gene expression analysis

Total RNA from monocytes stimulated with IgG was extracted using the RNeasy mini Kit (Qiagen) according to the manufacturer's instructions. Total RNA was treated with DNase I and used to obtained biotin labelled cRNA (Applied Biosystems). The quality and quantity of cRNA were determined using a NanoPhotometer^®^ (Implen). Gene expression profile was done using HumanHT-12 v4 bead chip array (Illumina) according to manufacturer's protocol. Groups of samples based on IgG source were equally distributed on the two chips to avoid batch effect.

Raw data was generated by Genome Studio and analyzed using R software 3.0.2 (limma and lme 4 packages). Background correction, quantile normalization and log 2 transformation were applied to standardized signal among samples. A linear mixed model (LMM) [17] and a finite mixture model (FMM) were applied to identify DE transcripts. A threshold >0.8 of the Bayesian posterior probability comparable to a 0.05 False Discovery Rate (FDR) threshold was chosen to reduce false positives.

A DV analysis as described in Ho et al. [13] was also performed. After outlier removal, an F test was applied to look for genes with significant DV. Multiple tests were corrected with a 0.05 FDR procedure.

Gene ontology (GO) enrichment was performed using Panther-GO (http://www.pantherdb.org/) and DAVID (https://david.ncifcrf.gov) bioinformatics tools. In the last case, main biological processes were clustered in a functional annotation analysis with a fold enrichment score based on Expression Analysis Systematic Explorer (EASE) score.

### 2.6 Validation of data by quantitative real-time PCR (qPCR)

Total RNA from monocytes stimulated with IgG was extracted using the RNeasy mini Kit (Qiagen) according to the manufacturer's instructions. cDNA was analyzed by quantitative RT-PCR using a TaqMan^®^ based assay (Applied Biosystems) in a DNA Engine Opticon continuous fluorescence detector (MJ Research). Gene expression was determined relative to GAPDH using the comparative threshold method. The inventoried Taqman probes used were as follows: PTK2B (Hs00169444_m1), TIPM2 (Hs00234278_m1), GP5 (Hs03027242_s1), FN1 (Hs01549976_m1), C4A (Hs00416393_g1), AKT1 (Hs00178289_m1), CAV1 (Hs00971716_m1), EPOR (Hs00959427_m1), NRP1 (Hs00826128_m1), CCL22 (Hs01574247_m1).

### 2.7 Statistical analysis

Statistical analyses were performed using GraphPad Prism software 5.0 (GraphPad Software). Data were first tested for normality and equal variance. If data were normally distributed, comparisons were made using one-way analysis of variance. If data was not normally distributed, a Kruskal-Wallis test was used.

## 3 Results

### 3.1 Clinical and laboratory characteristics of individuals

A total of 18 patients with APS (9 VT and 9 PM) and 9 HC subjects were included in the study (Table 1). Twenty-one (78%) of the subjects were female. Of the 18 patients with APS, 3 had SLE and 15 primary APS. Nine patients with VT+/PM- had thrombotic manifestations (6 venous, 6 arterial and 3 recurrent). Seven patients with VT-/PM+ had second trimester foetal losses and two a first trimester foetal loss. Serum and purified IgG from patients in the VT+/PM- and VT-/PM+ groups had higher levels of aCL and anti-β_2_GPI activity compared to HC, but these levels did not differ significantly between VT+/PM- and VT-/PM+ groups (Table 1).

**Table 1:** Clinical and laboratory features of patients and controls.

	Vascular Thrombosis (VT) (n = 9)	Pregnancy Morbidity (PM) (n = 9)	Healthy controls (HC) (n = 9)
Age (Mean ± SEM)	45.6 ± 4.9	39.7 ± 2.5	39.2 ± 5.0
Sex	6 F/3 M	9 F	6 F/3M
PAPS	7 (77.7%)	8 (88.8%)	0
SLE	2 (22.2%)	1 (11.1%)	0
Live births	6	12	7
Total APS-related PM	0	9 (2FT-PL,7ST-PL)	0
Vascular thrombosis	6 V, 6A, 3R	0	0
Serum LA-positive	7	8	NT
Serum aCL (Mean GPLU ± SEM)	160.6 ± 30.2	128.4 ± 17.3	7.7 ± 0.2
Serum anti-b_2_GPI (Mean GBU ± SEM)	68.6 ± 22.5	51.3 ± 18.8	3.9 ± 1.1
IgG aCL (Mean GPLU ± SEM)	84.0 ± 15.6	67.5 ± 19.3	5.9 ± 0.6
IgG anti-b_2_GPI (Mean GBU ± SEM)	39.1.±9.9	40.6 ± 13.2	4.8 ± 1.1
Other autoantibodies	anti-dsDNA 3, ANA 3	anti-dsDNA 1, ANA 1	0
Medication	OA 6, CS 1, IS 2	LDA 2, HCQ 1	0

Abbreviations: aCL, anti-cardiolipin antibodies; anti-β_2_GPI, anti-β_2_-glycoprotein-I antibodies; A, arterial; ANA, antinuclear antibodies; CS, corticosteroid; F, female; GPLU, IgG phospholipid units; GBU, IgG arbitrary units; HCQ, hydroxychloroquine; IS, Immunosuppressant; LDA, low dose aspirin; LA, lupus anticoagulant; M, male; NT, not tested; OA, oral anticoagulant; PAPS, primary antiphospholipid syndrome; PM, pregnancy morbidity; R, recurrent; SEM, standard error of the mean; SLE, systemic lupus erythematosus; ST-PL, second trimester pregnancy loss; TT-PL, third trimester pregnancy loss; V venous.

Necessarily, groups were not matched for gender as only women can suffer PM. Restriction of the experiment to using only samples from women was considered, but this would have made the results unrepresentative of the population of patients with APS, which includes both men and women. A statistical analysis of the microarray results stratified by gender rather than disease group was carried out and showed no clustering by gender.

### 3.2 Multidimensional scaling (MDS) analysis revealed high variability among samples

mRNA samples from monocytes of a single healthy individual treated with IgG from subjects in the following groups; VT+/PM- (n = 8), VT-/PM+ (n = 6) or HC (n = 8) were hybridized to Illumina oligonucleotide microarrays. Raw data included the expression of 24 arrays and 48203 probes. Arrays had a similar proportion of expressed probes (Welch's *t*-test = 0.7361; p-value = 0.4696). Following normalization and background correction, control probes were removed and 47316 probes were considered for further analysis.

**Fig. 1:**
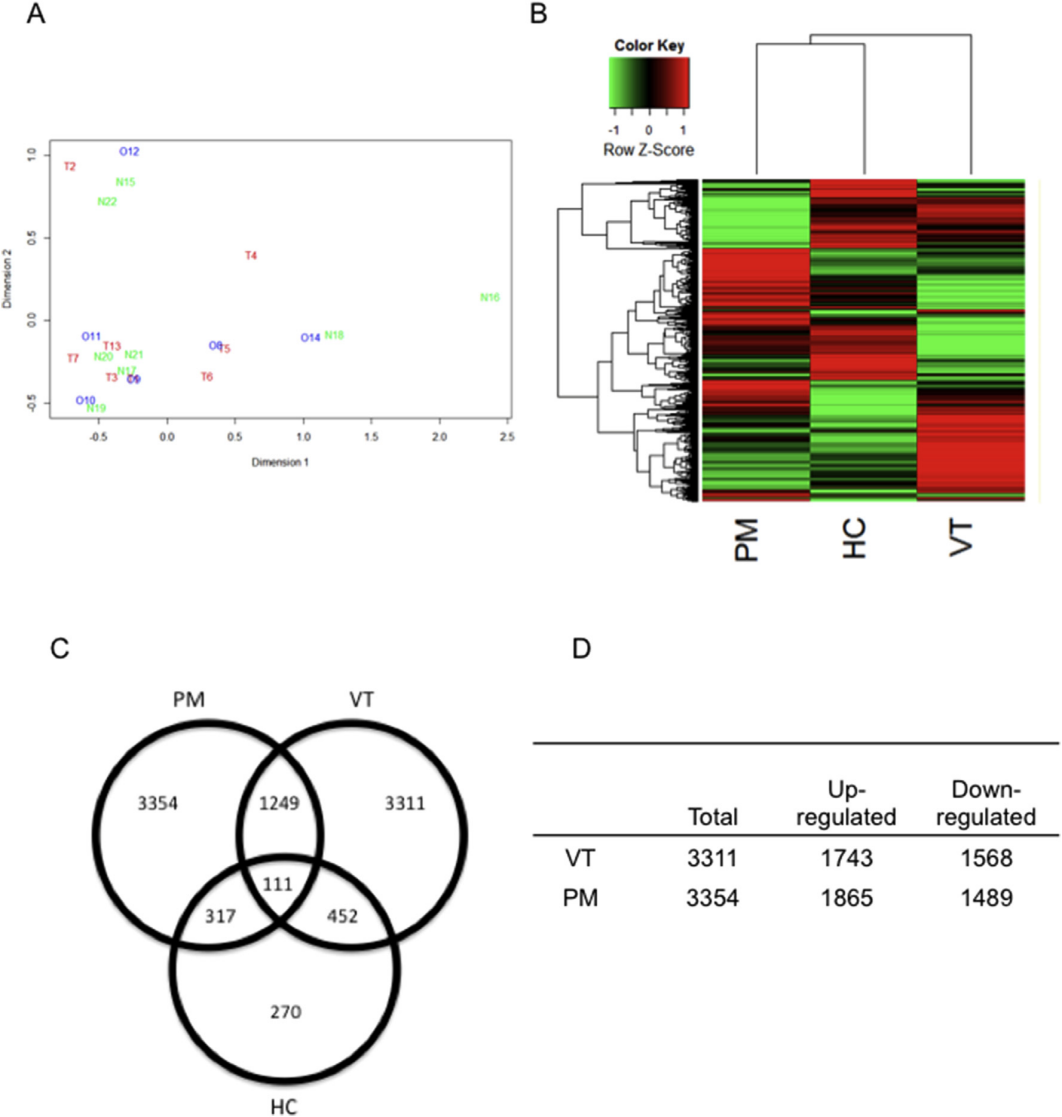
**Microarray analysis of human monocytes treated with VT**+**/PM-, VT-/PM** + **or HC-IgG.** (A) Multidimensional scaling (MDS) analysis of gene expression data. Treatments are shown by different colours and labels. T1-T7, T13 (red): VT+/PM-IgG; O8-O12, O14: VT-/PM+ IgG (blue); N15-N22 (green): HC- IgG. (B) Hierarchical clustering analysis of the DE probes in monocytes treated with VT+/PM-, VT-/PM+ or HC-IgG. Heat map of genes differentially expressed across the treatments. Each horizontal line represents a single transcript. Red and green denote high and low expression respectively. Dendrograms represent similarity of gene expression profile in the rows and treatments in the columns. (C) IgG from patients with VT+/PM-, VT-/PM+ differentially regulate gene expression in monocytes. Venn diagram illustrating the number of commonly and solely regulated genes in monocytes treated with VT+/PM-, VT-/PM+ or HC-IgG. The number of DE genes significantly regulated in monocytes treated with VT+/PM-, VT-/PM+ IgG is shown (D). (For interpretation of the references to colour in this figure legend, the reader is referred to the Web version of this article.)

MDS analysis was used to assess sample similarity based on pair-wise distances between samples. High response-to-treatment intra-variability among samples from the same group (monocytes exposed to VT+/PM- IgG, VT-/PM+ IgG or HC IgG) was observed (Fig. 1A). In order to interpret the data, a linear mixed model/finite mixture model was applied and differentially expressed transcripts (DE) in each treatment determined, resulting in a total of 12122 probes for analysis.

### 3.3 IgG from patients with VT-/PM+ or VT+/PM- induce distinct transcriptional profiles in monocytes

Global gene expression patterns of monocytes exposed to VT+/PM-, VT-/PM+ or HC-IgG were analyzed by hierarchical clustering (Fig. 1B). Genes were clustered according to their pattern of expression (up-regulation or down-regulation) in the vertical axes and by disease/control group (similarities in the overall gene expression profile) in the horizontal axes. The analysis revealed distinct and common gene expression patterns among the three groups and clearly defined genes whose expression was specific to VT+/PM-, VT-/PM+ or HC. The dendrogram separated the two APS sub-groups, clustering VT-/PM+ and HC-IgG together visibly apart from VT+/PM- IgG (Fig. 1B).

### 3.4 VT+/PM- and VT-/PM+ IgG differentially regulate gene expression in monocytes

DE was defined as a transcript showing an unusually high or low expression level under a particular treatment, compared with transcript expression levels of other genes under the same treatment. The Venn diagram in Fig. 1C shows the numbers of DE genes in monocytes exposed to each treatment. Thus, the circle on the left shows that 5031 genes were DE in monocytes exposed to VT-/PM+ IgG. Of these 5031 genes, 3354 were only DE in the VT-/PM+ group and not in monocytes exposed to VT+/PM- IgG or HC IgG. Similarly, 3311 genes were DE in the VT+/PM- IgG-exposed cells alone, whereas a much smaller number (270) were DE genes only in monocytes treated with HC-IgG. 1249 genes were DE in both APS subgroups but not HC.

Fig. 1D shows more detail of the 3354 genes that were DE only in the VT-/PM+ group - 1865 genes were up-regulated and 1489 were down-regulated. Similarly, of the 3311 genes that were only DE in the VT+/PM- IgG exposed cells. 1743 genes were up-regulated and 1568 down-regulated.

### 3.5 Functional analysis of DE genes revealed disease-specific genome signatures

**Fig. 2:**
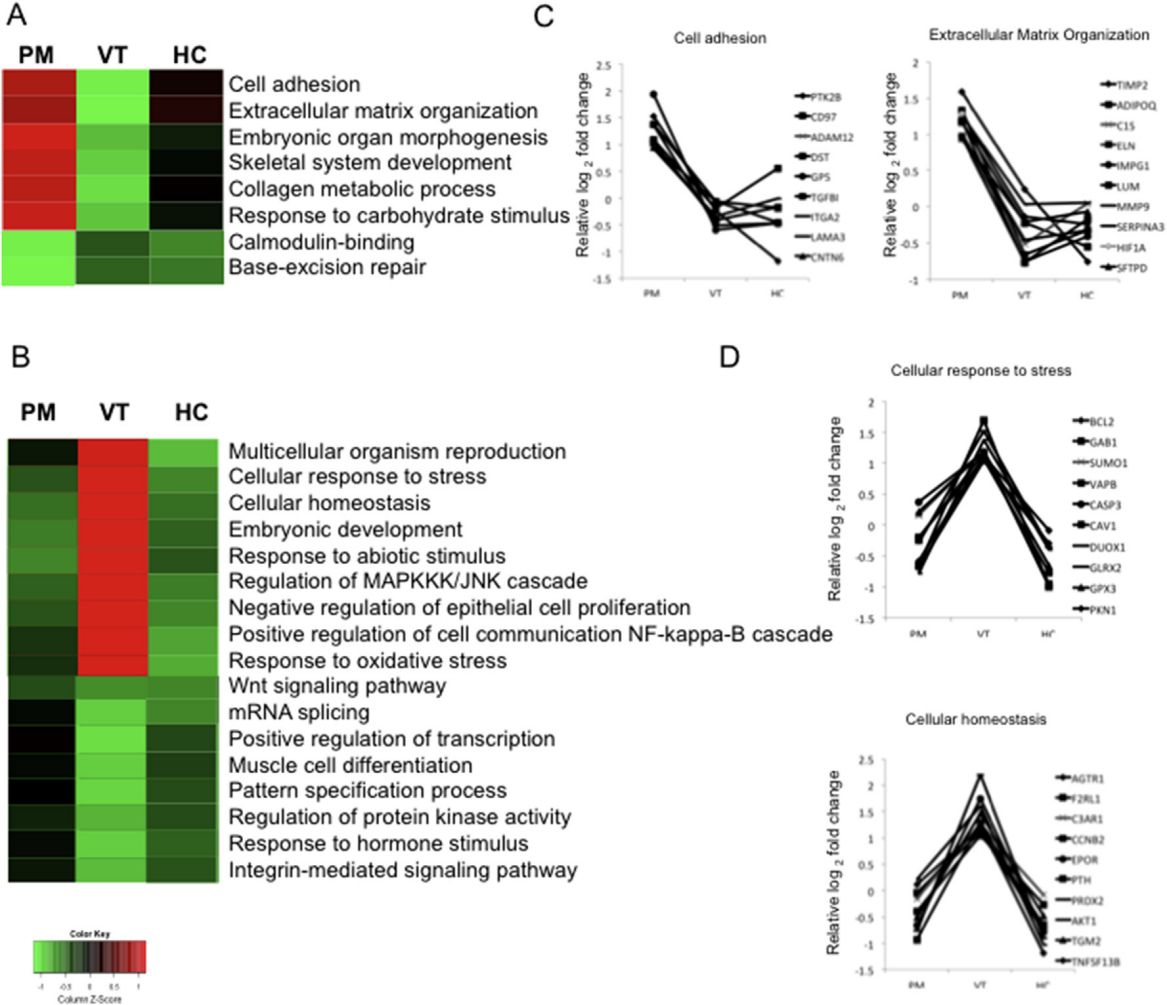
**Hierarchical clustering analysis of representative GO categories associated with VT**+**/PM-, VT-/PM**+ **IgG.** Heat map of overrepresented biological processes up-regulated and down-regulated in VT-/PM+ (A) and VT+/PM- (B) based on EASE score inferred from functional annotation clustering performed in DAVID. Red and green denote high and low expression respectively; black indicates no expression. Relative log_2_ fold change for 10 genes in top representative categories of up-regulated genes for VT-/PM+ (C) and VT+/PM- (D) are shown. (For interpretation of the references to colour in this figure legend, the reader is referred to the Web version of this article.)

**Supplementary Fig. 1:**
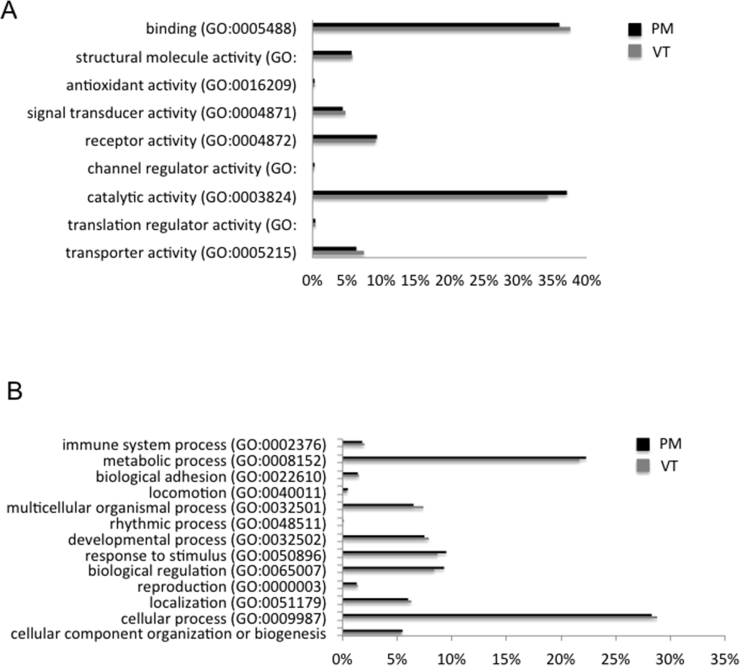
Functional classification of DE genes in monocytes exposed to VT-/PM+ and VT+/PM- IgG using Panther-GO (http://www.pantherdb.org/) analysis tool. Gene ontologies (GO) for Molecular function (A) and biological process (B) are shown.

Gene ontology analysis was performed to categorise DE genes in VT-/PM+ and VT+/PM- on the basis of their functional role and to identify disease-specific genome signatures. Preliminary analysis using Panther-GO (http://www.pantherdb.org/) revealed significant similarities in the most representative molecular function and biological processes categories (Supplementary Fig. 1). Deeper analysis using functional annotation clustering based on EASE score (https://david.ncifcrf.gov) was performed to examine overrepresented biological processes. Compared to VT+/PM- or HC, VT-/PM+ IgG showed specific up-regulation of genes involved in cell adhesion, extracellular matrix, embryonic morphogenesis, skeletal development and response to carbohydrate stimulus (Fig. 2A). The majority of these VT-/PM+ only GO categories are key processes known to be up-regulated during pregnancy and wound repair.

In contrast, VT+/PM- IgG specifically up-regulated genes involved in nine categories including multicellular organism reproduction, cell response to stress, cellular homeostasis, embryonic development, response to abiotic stimulus, regulation of the MAPKK kinase signalling, cell communication and oxidative stress (Fig. 2B). Overall these categories revealed that VT+/PM- induces a more versatile response to mediate immune functions.

Genes involved in Calcium binding and Wnt signalling pathways were amongst the categories specifically down-regulated by VT-/PM+ IgG and VT+/PM- IgG respectively (Fig. 2A and B). Examples of genes that are only up-regulated in monocytes exposed to VT-PM+ or VT+/PM- IgG are shown in Fig. 2C and D respectively. A more detailed list of the genes included in the functional categories overrepresented in monocytes exposed to VT-/PM+ and VT+/PM- IgG is shown in Supplementary Table 1.

### 3.6 Validation of selected DE genes by quantitative real time PCR (qPCR)

**Fig. 3:**
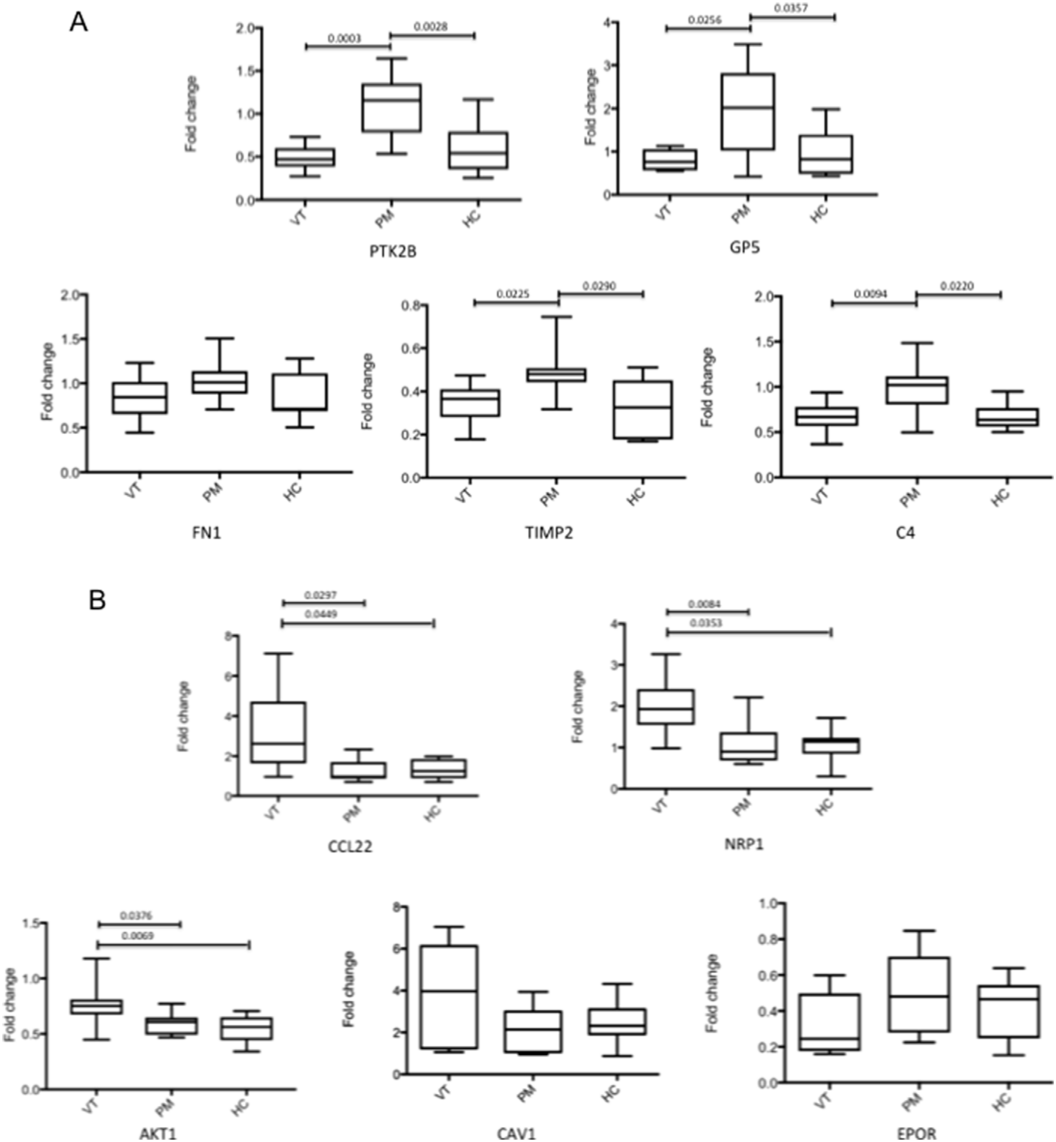
**Validation of microarray data by quantitative real time PCR (qPCR).***Ex-vivo* monocytes were treated with 200 μg/mL of individual IgG samples (9 VT+/PM-, 9 VT-/PM+ or 9 HC) for 6 h and levels of mRNA measured by qPCR. VT-/PM+ (A) and VT+/PM- (B) genes targets are shown. Data points represent the fold change expression of each sample compared to untreated; mean and standard errors are displayed. Data are representative of at least three independent experiments. Statistically significant difference was determined by one-way ANOVA, p values are displayed.

A set of DE genes with diverse levels of regulation and relevance to APS was selected for validation. *Ex-vivo* monocytes from a single healthy control were treated with individual VT+/PM- (n = 9), VT-/PM+ (n = 9) or HC (n = 9) IgG for 6 h. Levels of mRNA were measured by qPCR. Consistent with the microarray data, mRNA expression of Protein tyrosine kinase 2 beta (PTK2B), Glycoprotein V (platelet) (GP5), TIMP metallopeptidase inhibitor 2 (TIMP2) and Complement 4 (C4) were all significantly up-regulated in monocytes treated with VT-/PM+ IgG compared to VT+/PM- or HC-IgG (Fig. 3A). Although Fibronectin 1 (FN1) mRNA expression was induced by VT-/PM+ IgG compared to VT+/PM- or HC-IgG, these levels did not reach significance (Fig. 3A).

The mRNA expression of v-akt murine thymoma viral oncogene homolog 1 (AKT1), C-C motif chemokine 22 (CCL22) and Neuropilin 1 (NRP1) were significantly higher in monocytes treated with VT+/PM- IgG compared to VT-/PM+ or HC-IgG (Fig. 3B). The expression of Caveolin 1 (CAV1) mRNA was induced by VT+/PM- IgG compared to VT-/PM+ or HC-IgG, although these levels did not reach significance (Fig. 3B). Erythropoietin receptor (EPOR) mRNA expression however, was not found enriched in VT+/PM- IgG compared to either VT-/PM+ or HC-IgG.

### 3.7 Differential variability analysis identified some similar and some different functional gene signatures compared to DE analysis

**Fig. 4:**
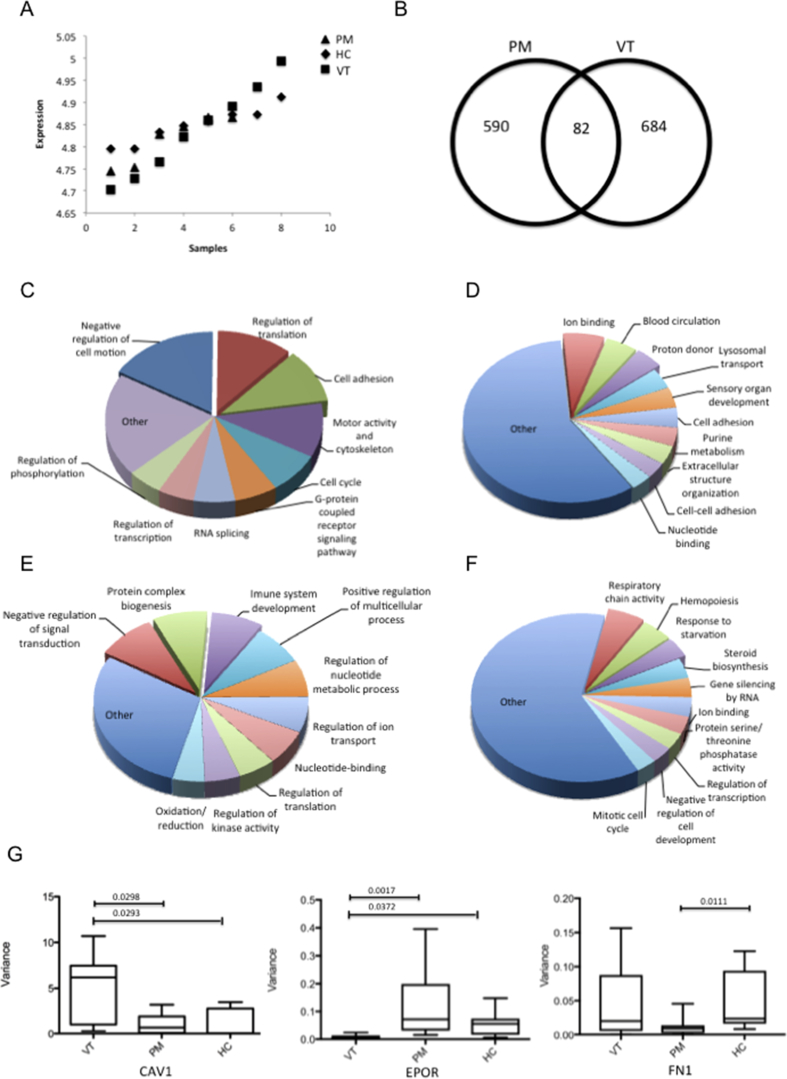
**Differential variability (DV) analysis.** An illustrative example of a differentially variable gene in VT+/PM- IgG compared to VT-/PM+ and HC-IgG is shown (A). Venn diagram displays the number of genes with differential variability expression in VT+/PM- and VT-/PM+ IgG compared to HC (B). Pie chart showing the GO analysis of biological processes distribution of genes with high (C) and low (D) variance in VT-/PM+ IgG and high and low variability in VT+/PM- IgG (E, F). Percentages were calculated as proportions of total EASE score. Exploded portions of the pie highlight the most representative categories. Validation of DV genes by qPCR (G), variance for each treatment was calculate and plotted; mean, standard errors and p values are displayed.

DV analysis aims to identify genes with a significant change in variance of expression between samples. Fig. 4A shows an example of a DV gene in VT+/PM- IgG compared to VT-/PM+ and HC-IgG. Transcripts were considered to have significant DV if the variance in the HC-IgG treated cells differed by a ratio of at least five-fold from that seen in the test group (VT+/PM- or VT-/PM+ IgG-exposed). In monocytes treated with VT-/PM+ IgG, 590 genes had DV compared to HC IgG (Fig. 4B). Of these, 177 genes displayed high variability and 413 had low variability. Similarly, 684 genes from VT+/PM-treated monocytes exhibited DV compared to HC, of which 162 and 522 genes had increased and decreased variance, respectively.

GO analysis of biologic processes using DAVID tools revealed that VT-/PM+ IgG had a significant effect increasing the variance of genes involved in the negative regulation of cell motion, regulation of translation and cell adhesion (Fig. 4C). VT-/PM+ IgG also significantly decreased the variance of genes associated with ion binding, blood circulation and proton donor (Fig. 4D).

Genes involved in negative regulation of signal transduction, protein complex biogenesis and immune system development had higher variance in VT+/PM- IgG-treated monocytes compared to HC (Fig. 4E). Respiratory chain activity, hemopoiesis and response to starvation were amongst the categories with low variance in VT+/PM- (Fig. 4F).

A more detailed list of the DV genes included in the functional categories for VT-/PM+ and VT+/PM- IgG is shown in Supplementary Table 2.

### 3.8 Validation of selected DV genes by quantitative real time PCR (qPCR)

To validate DV genes, *ex-vivo* monocytes from a single healthy control were treated with individual VT+/PM- (n = 9), VT-/PM+ (n = 9) or HC (n = 9) IgG for 6 h and mRNA expression of CAV1, FN1 and EPOR was measured. Variance for each of the treatments was calculated and plotted. As shown in Fig. 4G and in accordance with the DV analysis, CAV1 had significantly higher variability in VT+/PM- IgG compared to VT-/PM+ or HC IgG. In contrast, EPOR expression exhibited significantly decreased variance in VT+/PM-compared to VT-/PM+ or HC IgG. FN1 expression displayed significantly less variability in the presence of VT-/PM+ compared to HC IgG (Fig. 4G).

## 4 Discussion

We utilised gene expression profiling to identify and characterise different molecular pathways involved in the pathogenesis of thrombotic or obstetric APS. To our knowledge, this is the first study to combine two different approaches of gene expression analysis (DE and DV), to establish differences in gene expression resulting from the effect of VT+/PM- and VT-/PM+ APS-IgG on monocytes.

The analysis of gene expression profiles can be challenging due to the variability intrinsic to microarray data [18]. Investigations of the similarities in expression profiles of VT+/PM-, VT-/PM+ and HC IgG through MDS revealed high degree of variability in our samples. Excluding the major sources of experimental variability, individual sample variability seemed to be the main source of heterogeneity. APS itself is a heterogeneous disease and as reported in other human diseases, variability is in part due to the distinct evolution of individual response from the same disease subtype [19,20]. Furthermore, the use of polyclonal IgG from patients can represent a source of variability, because they contain a heterogeneous population of antibodies that may differ in their quantity, binding and ability to act upon cells.

In assessing microarray data it is important to utilize different methods of analysis and an appropriate statistical model, such as the linear mixed model used here. This approach can help to overcome variability between samples. Indeed, LMM considers a global model that includes variance heterogeneity [17]. DV analysis also represents a meaningful alternative to overcome heterogeneity as it aims to identify genes with a significant change in variance of expression between different groups. A change in variability may reveal a more irregular expression pattern. Biologically relevant genes can be DV without being DE [13]. Consistent with this, we identified a smaller number of DV genes compared to DE genes, the number of up-regulated and down-regulated DE were similar, whereas DV genes with decreased variability predominated in APS samples. Also, although there was an overlap between the genes identified by DV and DE, as expected different transcripts were determined in each analysis. It was of particular interest that three genes (FN1, CAV-1 and EPOR) were identified as important by both DE and DV analysis but qPCR validation confirmed the DV results rather than the DE results.

Our microarray analysis identified and validated targets found in genomics and classical proteomics APS studies in monocytes [8,12], peripheral blood mononuclear cells (PBMCs) [10], endothelial cells [11,21], as well as markers associated with pregnancy complications in the presence [22,23] and absence of APS [24,25].

### 4.1 Genes that are potentially important in obstetric APS, based on DE and DV results in monocytes exposed to VT-/PM+ IgG

According to the DE analysis, VT-PM+ IgG induced the expression of several genes involved in cell adhesion, extracellular matrix (ECM), embryonic morphogenesis and skeletal development. These processes are critical for developmental events that take place during pregnancy. The adhesion of cells to each other, to other cell types and to ECM is a crucial part of fetal development and relies on the expression of cell adhesion molecules and their ligands. As reviewed by Rozario et al. [26], ECM is highly modified during fetal development and its interaction with integrins facilitates cell adhesion and migration. Cell signalling through ECM can impact cell fate decisions, cell proliferation and survival, and other specialized functions.

Consistent with our findings, several pregnancy-associated disorders including pre-eclampsia, fetal growth retardation and miscarriage have been linked to abnormalities in expression of particular cell adhesion molecules (CAMs) and/or their ECM ligands [23,27,28]. Another distinguishing feature of the results for VT/-PM+ IgG is the expression of genes that change the state or activity of a cell as a result of a carbohydrate stimulus, suggesting an increased metabolic activity in monocytes in the presence of VT-/PM+ aPL.

TIMP2 is a target identified in the VT-/PM+ IgG-exposed cells. Trophoblast migration and invasion are regulated by the balance between the production of the matrix metalloproteinases and their tissue inhibitors, TIMP1 and TIMP2. aPL induced the secretion of TIMP2 diminishing the ability of trophoblast to migrate [29]. Consistent with this, we have previously showed that purified polyclonal IgG from pregnancy morbidity but not vascular thrombosis was able to inhibit the migration of human trophoblast cells [30].

Anti- β2GPI also disrupt the balance of trophoblast angiogenic factors inducing the secretion of VEGF through a TLR4 independent response, most likely mediated by TLR8 or IL-1R [31,32]. In this study, we found VEGF, TLR8 and IL-1R were specifically induced in the presence of VT-/PM+ IgG.

Activation of complement mediates fetal damage in both mouse models and patients with obstetric APS [33,34]. Blockade of classical and alternative pathways prevents growth restriction and pregnancy loss. We identified two classical pathway complement components exclusively induced by VT-/PM+ IgG; C4A, and C1s.

GP5, a constituent of the receptor for von Willebrand factor, was induced by VT-/PM+ IgG. A report by Shi et al. [35] shows that anti-β2GPI antibodies can bind glycoprotein Ib-IX-V leading to platelet activation. Interestingly, activated platelets have been implicated in the pathogenesis of hypertensive disorders of pregnancy through their ability to propagate endothelial dysfunction [36]. Platelet activation may directly contribute to placental thrombosis and dysfunction that is a feature of recurrent miscarriage but not specific to APS [37].

Both DV and DE analysis in this study identified fibronectin as being differentially regulated in monocytes exposed to VT-/PM+ IgG. Fibronectin is essential for embryogenesis, cell adhesion and growth [38]. Small cohort studies have reported anti-fibronectin antibodies to be present in 34% patients with SLE and to be correlated with musculoskeletal disease activity [39]. In addition, aPL have been reported to increase the expression of fibronectin in a study of endothelial cell dysfunction in cardiac valvulopathy in APS [40]. There is a link between this target and pregnancy morbidity, as fetal fibronectin appears to be a promising marker for both preterm delivery and morbidity in twin gestation [41].

### 4.2 Genes that are potentially important in thrombotic APS, based on DE and DV results in monocytes exposed to VT+/PM- IgG

Our results in monocytes exposed to VT+/PM- IgG were consistent with previous findings. There were significant overlaps between our results and those of Perez-Sanchez et al. [12] in their microarray study of monocytes derived from patients with APS. In particular, the DE analysis in both studies identified genes involved in cell response to stress, cellular homeostasis and oxidative stress. These findings are consistent with the known importance of these cellular pathways in the activation of various cell types, in the pathogenesis of thrombosis and cardiovascular disease [42]. Genes such as TLR4, Annexin II, VEGF, TGFβ, C3R, PKC, PKN, BCL2 as well as many genes involved in mitochondrial function and oxidative stress like MPO, GPX3, DUOX1, UCP3 were common to both our results and the previous studies of Lopez-Pedrera's group [8,12]. In addition, we confirmed our previous findings comparing the intracellular effects of VT+/PM- and VT-/PM+ IgG in monocytes [7]. This microarray analysis found the regulation of MAPKK kinase signalling and cell communication through Ikappa kinase/NF-kappa-B cascade to be a distinctive feature of VT+/PM- IgG.

VT+/PM-IgG also up-regulated genes involved with multicellular organism reproduction and embryonic development. The majority of the genes represented in both categories are related to cell cycle, proliferation and differentiation, suggesting VT+/PM-IgG may induce a survival signal in monocytes. Indeed AKT, a well-known marker of proliferation, was one of the top up-regulated genes in the presence of VT+/PM-IgG. Canaud et al. [43] reported the stimulation of AKT through the mTORC pathway in endothelial cells exposed to IgG from patients with APS. AKT activation was associated with vascular proliferation and vascular injury in patients with APS nephropathy.

Our data showed that VT+/PM-IgG might drive the production of pro-inflammatory cytokines and chemokines downstream of the activation of TLR4 and MAPK signalling pathways. A number of interleukins and their receptors, chemokine receptors, interferons, and genes belonging to the tumour necrosis factor pathway were solely expressed in the presence of VT+/PM- IgG. The expression of novel inflammatory chemokines like CCL22 that had not previously been associated with APS was shown and validated in this study.

Up-regulation of the expression of genes involved in complement and coagulation cascade; C3, C3R, C5, C1S, CD46, coagulation factor II, FGG and protein S was seen in monocytes exposed to VT+/PM- IgG. Within this category CAV-1, an integral membrane protein, not previously linked to APS, has a critical role in the regulation of tissue factor pathway inhibitor (TFPI) and the extrinsic pathway of coagulation [44].

In addition to the coagulation cascade, VT+/PM- IgG induced genes involved in angiogenesis, including NRP1 that binds and regulates vascular endothelial growth factor (VEGF). Given that aPL have previously been shown to be associated with an inhibition of angiogenesis driving defective placentation in the APS [45], it is interesting that we found NRP1 to be down-regulated in monocytes treated with VT-/PM+ IgG compared to.

Overall, it is possible that VT-/PM+ and VT+/PM- IgG trigger the expression of distinctive pathways as they might bind to different antigens/receptors on the surface of monocytes. Also, depending on their affinity and avidity these autoantibodies can either activate or inhibit specific cellular responses. Similarly, it is likely that VT-/PM+ and VT+/PM- IgG differ in the proportion of IgG isotypes in particular subclasses (IgG1, IgG2, IgG 3 and IgG4) with diverse abilities to interact with Fc receptors and to elicit particular monocyte functions.

In conclusion, this is the first microarray study that directly compares the effects of IgG from patients with vascular thrombosis and pregnancy morbidity on human monocytes. The identification of different genes and functional categories support the hypothesis that VT+/PM- IgG and VT-/PM+ IgG trigger distinctive physiological pathways in monocytes. This finding might contribute to our understanding of the pathology of APS and to the development of different targeted therapies for thrombotic and obstetric APS.

## Declaration of interest

None.

## Conflict of interest disclosures

None.

## Figures and Tables

**Supplementary Table 1:**
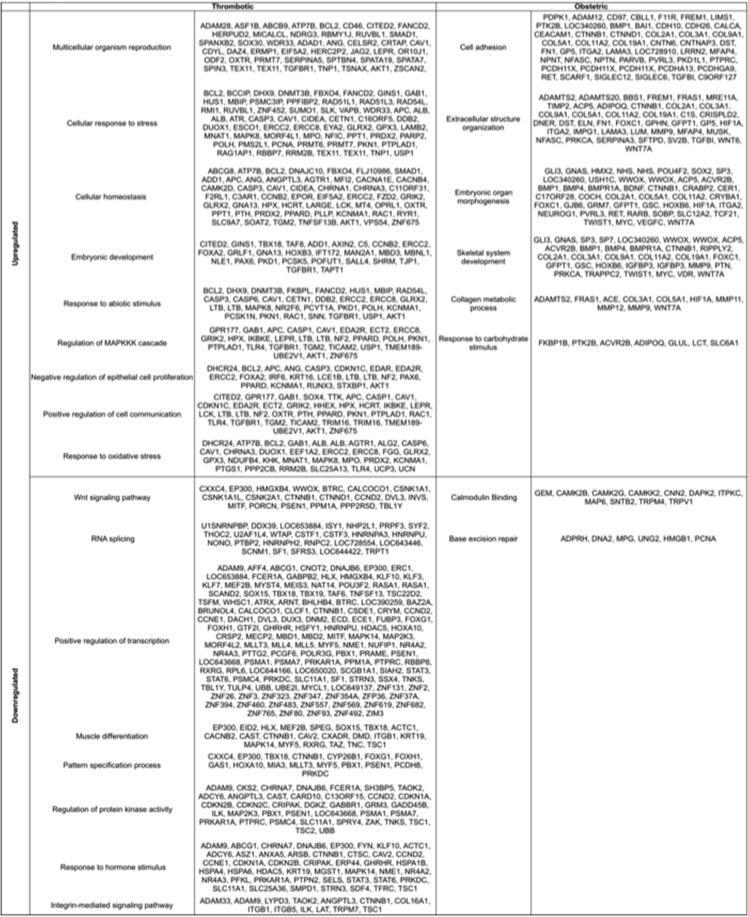
List of DE genes included in the GO in the functional categories overrepresented in monocytes exposed to VT-/PM+ and VT+/PM- IgG.

**Supplementary Table 2:**
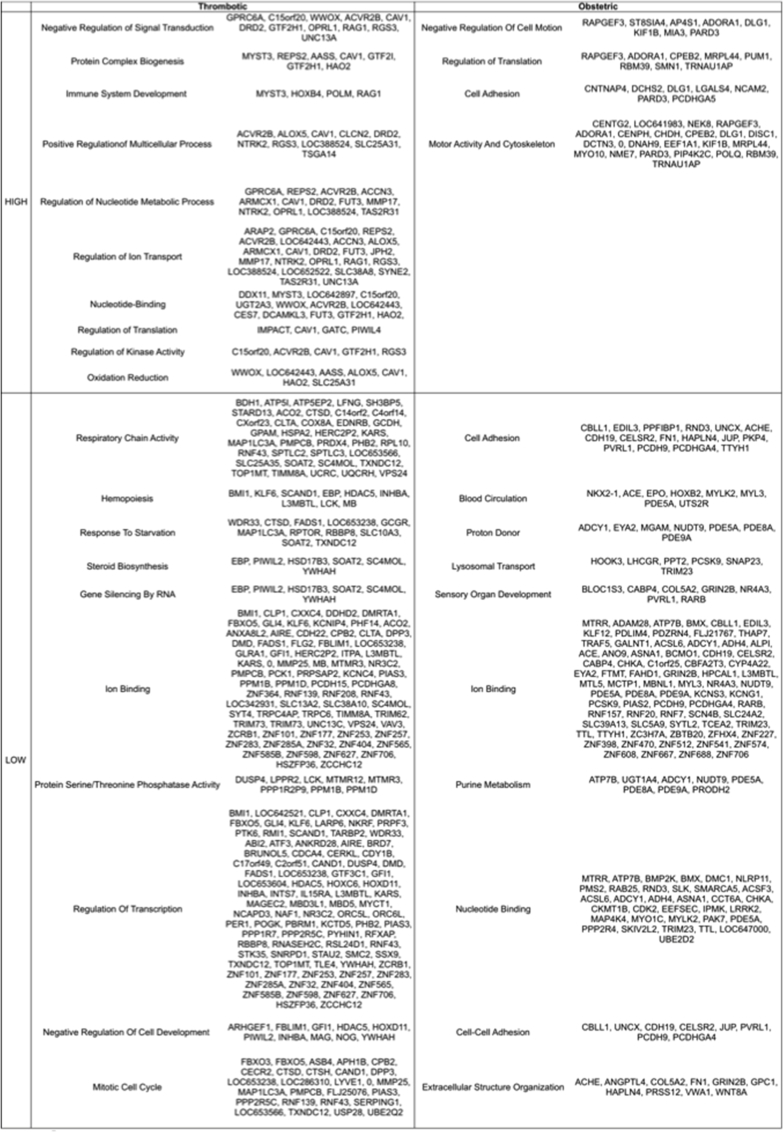
List of the DV genes included in the functional categories for VT-/PM+ and VT+/PM- IgG.

## References

[1] Alijotas-Reig J., Ferrer-Oliveras R., Ruffatti A., Tincani A., Lefkou E., Bertero M.T. (2015). The European registry on obstetric antiphospholipid syndrome (EUROAPS): a survey of 247 consecutive cases. Autoimmun. Rev..

[2] Cervera R., Serrano R., Pons-Estel G.J., Ceberio-Hualde L., Shoenfeld Y., de Ramon E. (2015). Morbidity and mortality in the antiphospholipid syndrome during a 10-year period: a multicentre prospective study of 1000 patients. Ann. Rheum. Dis..

[3] Lockshin M.D., Kim M., Laskin C.A., Guerra M., Branch D.W., Merrill J. (2012). Prediction of adverse pregnancy outcome by the presence of lupus anticoagulant, but not anticardiolipin antibody, in patients with antiphospholipid antibodies. Arthritis Rheum..

[4] Cuadrado M.J., Buendia P., Velasco F., Aguirre M.A., Barbarroja N., Torres L.A. (2006). Vascular endothelial growth factor expression in monocytes from patients with primary antiphospholipid syndrome. J. Thromb. Haemostasis.

[5] Lopez-Pedrera C., Aguirre M.A., Buendia P., Barbarroja N., Ruiz-Limon P., Collantes-Estevez E. (2010). Differential expression of protease-activated receptors in monocytes from patients with primary antiphospholipid syndrome. Arthritis Rheum..

[6] Lopez-Pedrera C., Buendia P., Cuadrado M.J., Siendones E., Aguirre M.A., Barbarroja N. (2006). Antiphospholipid antibodies from patients with the antiphospholipid syndrome induce monocyte tissue factor expression through the simultaneous activation of NF-kappaB/Rel proteins via the p38 mitogen-activated protein kinase pathway, and of the MEK-1/ERK pathway. Arthritis Rheum..

[7] Lambrianides A., Giles I.P. (2010). Use of monoclonal antibodies to dissect specificity and pathogenesis of antiphospholipid antibodies. Lupus.

[8] Lopez-Pedrera C., Cuadrado M.J., Herandez V., Buendia P., Aguirre M.A., Barbarroja N. (2008). Proteomic analysis in monocytes of antiphospholipid syndrome patients: deregulation of proteins related to the development of thrombosis. Arthritis Rheum..

[9] Ripoll V.M., Lambrianides A., Pierangeli S.S., Poulton K., Ioannou Y., Heywood W.E. (2014). Changes in regulation of human monocyte proteins in response to IgG from patients with antiphospholipid syndrome. Blood.

[10] Bernales I., Fullaondo A., Marin-Vidalled M.J., Ucar E., Martinez-Taboada V., Lopez-Hoyos M. (2008). Innate immune response gene expression profiles characterize primary antiphospholipid syndrome. Gene Immun..

[11] Hamid C., Norgate K., D'Cruz D.P., Khamashta M.A., Arno M., Pearson J.D. (2007). Anti-beta2GPI-antibody-induced endothelial cell gene expression profiling reveals induction of novel pro-inflammatory genes potentially involved in primary antiphospholipid syndrome. Ann. Rheum. Dis..

[12] Perez-Sanchez C., Barbarroja N., Messineo S., Ruiz-Limon P., Rodriguez-Ariza A., Jimenez-Gomez Y. (2015). Gene profiling reveals specific molecular pathways in the pathogenesis of atherosclerosis and cardiovascular disease in antiphospholipid syndrome, systemic lupus erythematosus and antiphospholipid syndrome with lupus. Ann. Rheum. Dis..

[13] Ho J.W., Stefani M., dos Remedios C.G., Charleston M.A. (2008). Differential variability analysis of gene expression and its application to human diseases. Bioinformatics.

[14] Miyakis S., Lockshin M.D., Atsumi T., Branch D.W., Brey R.L., Cervera R. (2006). International consensus statement on an update of the classification criteria for definite antiphospholipid syndrome (APS). J. Thromb. Haemostasis.

[15] Tan E.M., Cohen A.S., Fries J.F., Masi A.T., McShane D.J., Rothfield N.F. (1982). The 1982 revised criteria for the classification of systemic lupus erythematosus. Arthritis Rheum..

[16] Giles I., Lambrianides N., Pattni N., Faulkes D., Latchman D., Chen P. (2006). Arginine residues are important in determining the binding of human monoclonal antiphospholipid antibodies to clinically relevant antigens. J. Immunol..

[17] Trabzuni D., United Kingdom Brain Expression C., Thomson P.C. (2014). Analysis of gene expression data using a linear mixed model/finite mixture model approach: application to regional differences in the human brain. Bioinformatics.

[18] Hatfield G.W., Hung S.P., Baldi P. (2003). Differential analysis of DNA microarray gene expression data. Mol. Microbiol..

[19] Bravo H.C., Pihur V., McCall M., Irizarry R.A., Leek J.T. (2012). Gene expression anti-profiles as a basis for accurate universal cancer signatures. BMC Bioinf..

[20] MacDonald J.W., Ghosh D. (2006). COPA-cancer outlier profile analysis. Bioinformatics.

[21] Grenn R.C., Yalavarthi S., Gandhi A.A., Kazzaz N.M., Nunez-Alvarez C., Hernandez-Ramirez D. (2017). Endothelial progenitor dysfunction associates with a type I interferon signature in primary antiphospholipid syndrome. Ann. Rheum. Dis..

[22] Alvarez A.M., Neubeck S., Parra S., Markert U.R., Cadavid A.P. (2017). Serum protein profile in women with pregnancy morbidity associated with antiphospholipid syndrome. J. Hum. Reprod. Sci..

[23] Pantham P., Rosario R., Chen Q., Print C.G., Chamley L.W. (2012). Transcriptomic analysis of placenta affected by antiphospholipid antibodies: following the TRAIL of trophoblast death. J. Reprod. Immunol..

[24] Carty D.M., Delles C., Dominiczak A.F. (2008). Novel biomarkers for predicting preeclampsia. Trends Cardiovasc. Med..

[25] Leavey K., Bainbridge S.A., Cox B.J. (2015). Large scale aggregate microarray analysis reveals three distinct molecular subclasses of human preeclampsia. PLoS One.

[26] Rozario T., DeSimone D.W. (2010). The extracellular matrix in development and morphogenesis: a dynamic view. Dev. Biol..

[27] Lyall F., Greer I.A., Boswell F., Young A., Macara L.M., Jeffers M.D. (1995). Expression of cell adhesion molecules in placentae from pregnancies complicated by pre-eclampsia and intrauterine growth retardation. Placenta.

[28] Othman R., Omar M.H., Shan L.P., Shafiee M.N., Jamal R., Mokhtar N.M. (2012). Microarray profiling of secretory-phase endometrium from patients with recurrent miscarriage. Reprod. Biol..

[29] Albert C.R., Schlesinger W.J., Viall C.A., Mulla M.J., Brosens J.J., Chamley L.W. (2014). Effect of hydroxychloroquine on antiphospholipid antibody-induced changes in first trimester trophoblast function. Am. J. Reprod. Immunol..

[30] Poulton K., Ripoll V.M., Pericleous C., Meroni P.L., Gerosa M., Ioannou Y. (2015). Purified IgG from patients with obstetric but not IgG from non-obstetric antiphospholipid syndrome inhibit trophoblast invasion. Am. J. Reprod. Immunol..

[31] Fitzgerald J.S., Poehlmann T.G., Schleussner E., Markert U.R. (2008). Trophoblast invasion: the role of intracellular cytokine signalling via signal transducer and activator of transcription 3 (STAT3). Hum. Reprod. Update.

[32] Mulla M.J., Salmon J.E., Chamley L.W., Brosens J.J., Boeras C.M., Kavathas P.B. (2013). A role for uric acid and the Nalp3 inflammasome in antiphospholipid antibody-induced IL-1 beta production by human first trimester trophoblast. PLoS One.

[33] Cohen D., Buurma A., Goemaere N.N., Girardi G., le Cessie S., Scherjon S. (2011). Classical complement activation as a footprint for murine and human antiphospholipid antibody-induced fetal loss. J. Pathol..

[34] Shamonki J.M., Salmon J.E., Hyjek E., Baergen R.N. (2007). Excessive complement activation is associated with placental injury in patients with antiphospholipid antibodies. Am. J. Obstet. Gynecol..

[35] Shi T., Giannakopoulos B., Yan X., Yu P., Berndt M.C., Andrews R.K. (2006). Anti-beta2-glycoprotein I antibodies in complex with beta2-glycoprotein I can activate platelets in a dysregulated manner via glycoprotein Ib-IX-V. Arthritis Rheum..

[36] Nadar S., Lip G.Y. (2004). Platelet activation in the hypertensive disorders of pregnancy. Expet Opin. Invest. Drugs.

[37] Sebire N.J., Backos M., El Gaddal S., Goldin R.D., Regan L. (2003). Placental pathology, antiphospholipid antibodies, and pregnancy outcome in recurrent miscarriage patients. Obstet. Gynecol..

[38] Mao Y., Schwarzbauer J.E. (2005). Fibronectin fibrillogenesis, a cell-mediated matrix assembly process. Matrix Biol..

[39] Atta M.S., Powell R.J., Hopkinson N.D., Todd I. (1994). Human anti-fibronectin antibodies in systemic lupus erythematosus: occurrence and antigenic specificity. Clin. Exp. Immunol..

[40] Afek A., Shoenfeld Y., Manor R., Goldberg I., Ziporen L., George J. (1999). Increased endothelial cell expression of alpha3beta1 integrin in cardiac valvulopathy in the primary (Hughes) and secondary antiphospholipid syndrome. Lupus.

[41] Kiefer D.G., Vintzileos A.M. (2008). The utility of fetal fibronectin in the prediction and prevention of spontaneous preterm birth. Rev Obstet Gynecol.

[42] Madamanchi N.R., Hakim Z.S., Runge M.S. (2005). Oxidative stress in atherogenesis and arterial thrombosis: the disconnect between cellular studies and clinical outcomes. J. Thromb. Haemostasis.

[43] Canaud G., Bienaime F., Tabarin F., Bataillon G., Seilhean D., Noel L.H. (2014). Inhibition of the mTORC pathway in the antiphospholipid syndrome. N. Engl. J. Med..

[44] Lupu C., Hu X., Lupu F. (2005). Caveolin-1 enhances tissue factor pathway inhibitor exposure and function on the cell surface. J. Biol. Chem..

[45] Di Simone N., Marana R., Castellani R., Di Nicuolo F., D'Alessio M.C., Raschi E. (2010). Decreased expression of heparin-binding epidermal growth factor-like growth factor as a newly identified pathogenic mechanism of antiphospholipid-mediated defective placentation. Arthritis Rheum..

